# The effect of circadian on the productivity of nurses with the mediating role of quality of work life

**DOI:** 10.1186/s12912-024-01746-x

**Published:** 2024-02-02

**Authors:** Yasaman Poormoosa, Mohammad Amerzadeh, Ahad Alizadeh, Rohollah Kalhor

**Affiliations:** 1 Student Research Committee, School of Public Health, Qazvin University of Medical Sciences, Qazvin, Iran; 2 Metabolic Diseases Research Center, Research Institute for Prevention of Non-Communicable Diseases, , Qazvin University of Medical Sciences, Qazvin, Iran; 3 Medical Microbiology Research Center, , Qazvin University of Medical Sciences, Qazvin, Iran; 4 School of Public Health , Qazvin University of Medical Sciences, Qazvin, Iran

**Keywords:** Shift work, Circadian, Nurses, Efficiency, Quality of work life

## Abstract

**Background:**

Circadian rhythms, as an integral part of daily life, govern the scheduling, management, and coordination of living organisms. Given the irregular nature of shift patterns in nurses’ work schedules, investigating their implications is paramount to increasing Quality of Work Life (QWL) and productivity. The study aimed to investigate the impact of circadian rhythm on the efficiency of nurses working in hospitals in Qazvin, Iran, with QWL serving as a mediating variable.

**Methods:**

This study employed a descriptive-analytical research design, utilizing cross-sectional data collected in 2022–2023 based on the implementation of Structural Equation Modeling (SEM). The number of participants was 378 nurses. The data were obtained by administering a questionnaire and various tools, organized into four sections: demographic information, the Circadian Questionnaire, the Quality of Work Life Questionnaire, and the Nurses’ Efficiency Questionnaire. The collected data were subsequently analyzed using SEM techniques within the R software.

**Results:**

The findings demonstrated statistically significant variations in mean scores about gender and efficiency (*p* = 0.008), marital status and efficiency (*p* = 0.000), and employment type and efficiency (*p* = 0.002) among the study participants. There was a significant association between shift patterns and QWL (*p* = 0.004). Expressly, the confirmed results indicated a direct impact of circadian on QWL (with a path coefficient of 0.013), as well as an indirect impact on efficiency mediated by the variable QWL (with a path coefficient of 0.037) (*p* < 0.05).

**Conclusion:**

Due to the critical role of nurses in the healthcare system, implementing strategies that promote their efficiency is paramount. Therefore, managers can create an environment that enhances nurses’ productivity by improving methods that positively impact their QWL.

## Background

The nocturnal time scheduling system is crucial in coordinating various daily processes, including the daily rhythm of glucose metabolism in humans. The central clock regulates the body’s overall response to food intake, energy expenditure, and insulin sensitivity, while local clocks further regulate these actions. Disharmony among the nocturnal time scheduling system components and daily rhythms of sleep-wake behavior or food intake, resulting from genetic, environmental, or behavioral factors, can contribute to insulin resistance [1].

When circadian rhythms are disrupted or desynchronized due to jet lag, shift work, or other lifestyle factors, adverse health consequences arise, and the risk of diseases, such as cardiovascular diseases or metabolic disorders, increases. Although the negative impact of circadian rhythm disruption has been well-documented, the utilization or manipulation of biological timing for health benefits has been overlooked [2].

Shift work is associated with decreased melatonin levels and disruption of circadian rhythms [3]. Rotating shifts lead to increased sleep disturbances, fatigue, an increased risk of gastrointestinal disorders, short-term neurological impairments, and an increased susceptibility to obesity and diabetes [4]. Shift work is accompanied by impaired immune function, and shift workers often have a weakened immune system [5]. Furthermore, irregular working hours can result in adverse health outcomes, such as unhealthy dietary habits, smoking, and sedentary behavior. Non-compliance with shift schedules can lead to occupational burnout and decreased performance among individuals [6]. This disruption of the natural circadian rhythm can also impact individuals’ social and familial relationships due to psychological changes [7]. In the long term, shift work may increase the risk of mental disorders, particularly depression and anxiety [8]. Despite the numerous adverse effects of shift work on human health, population migration to cities, industrial development, and economic needs have increased shift-based occupations [4].

There have been multiple definitions of Quality of Work Life (QWL), which include factors and conditions related to the work environment, as well as employees’ attitudes and feelings about their work. Improving QWL leads to higher employee performance and productivity and significantly affects their mental health [9, 10]. Among the factors contributing to a decrease in the QWL of nurses, one can mention factors such as excessive workload, job-related stress and tension, fatigue, and insufficient leisure time [11–13].

Human resource productivity in organizations refers to the optimal and desirable utilization of employees’ talents and abilities. Improving productivity is one of the important goals of any organization [14, 15]. Enhancing the productivity of nurses serves as an assessment criterion for continuous improvement for all components of a hospital and leads to improved quality in all services [14].

Improving QWL directly affects individual satisfaction, which in turn influences the performance and productivity of employees [16]. Since the work of nurses involves the care of human lives, implementing organized and ergonomic planning methods can minimize harm to the nurses’ health and positively impact their efficiency and effectiveness in patient care [17]. QWL among nurses can directly and indirectly impact patient safety and the quality of care provided to patients as well [18].

Considering that nurses constitute a significant portion of the healthcare workforce, efforts to improve work schedules and shifts according to the circadian rhythm can potentially significantly impact the QWL and, consequently, their productivity. Besides, QWL is a comprehensive program that can lead to improved performance of employees within organizations and, consequently, their satisfaction. It promotes motivation and enhances individuals’ morale, driving them towards better performance. Moreover, professional nurses are responsible for the quality of life of patients. Therefore, before assisting patients, they must have a conducive QWL [19].

Based on the findings of numerous studies, improving nurses’ QWL increases their productivity [20–22]. Typically, job rotation, ergonomic considerations, and nurses’ health are not considered in scheduling and shift planning. Neglecting these factors leads to physical and mental harm and, consequently, job dissatisfaction among nurses, resulting in a decline in their QWL [23]. Given the diverse and irregular patterns of shift work in nurses’ work schedules, exploring their impact could prove highly impactful in optimizing the implementation of rotational shifts, thereby promoting QWL and enhancing productivity. This study aimed to investigate the impact of circadian rhythm on the productivity of nurses working in educational hospitals in Qazvin, Iran, with QWL playing a mediating role.

Research questions:

1. What is the status of circadian rhythm among nurses in educational hospitals of Qazvin?
2. What is the status of QWL among nurses in educational hospitals of Qazvin?
3. What is the productivity status among nurses in Qazvin’s educational hospitals?

Research Hypotheses:

1. There is a relationship between high-quality circadian rhythm and QWL among nurses in educational hospitals of Qazvin.
2. There is a relationship between circadian rhythm and nurses’ productivity in Qazvin educational hospitals.
3. There is a relationship between QWL and nurses’ productivity in Qazvin educational hospitals.
4. QWL mediates the relationship between circadian rhythm and productivity among nurses in educational hospitals of Qazvin.

## Methods

### Study design

This study employed a descriptive-analytical research design, utilizing cross-sectional data. We used the STROBE cross-sectional reporting guidelines. It was conducted from 2022 to 2023 among nurses in educational hospitals affiliated with Qazvin University of Medical Sciences (QUMS). After obtaining permission and explaining the research objectives and how to complete the questionnaire, the participants completed the questionnaire sheets at their workplace. Subsequently, the questionnaires were collected, and after ensuring that all questions were answered, they were entered into the software for analysis.

### Participants and recruitment

The study population consisted of all employed nurses in the educational hospitals of Qazvin City, which included six hospitals (Rajaei, Kowsar, Bu Ali, Ghods, Vali-Asr, and 22 Bahman). The total number of employed nurses in these educational centers was 1,313 individuals.

For Structural Equation Modeling (SEM) studies, the minimum sample size is five samples per item, and the maximum is 15. Considering the 67 items in this study, the required sample size ranged from 335 to 1005. However, due to the limited population size (1,313 nurses), the sample size was determined using the Morgan table, resulting in a final sample size of 302, considering a 30% drop rate, which accounted for 393 questionnaires. The hospitals of Rajaei, Kowsar, Bu Ali, Ghods, Vali-Asr, and 22 Bahman had sample sizes of 65, 29, 117, 49, 102, and 16, respectively. After distributing 393 questionnaires, approximately 378 completed questionnaires were collected (a response rate of 96%) and entered into statistical software for analysis. A stratified sampling approach was adopted to achieve the desired sample, whereby the number of individuals in each stratum was ascertained. Subsequently, a systematic random sampling technique was employed within each stratum to select the participants.

#### Inclusion criteria

- Nurses who expressed a willingness to participate in the study.
- Nurses with a minimum of one year of work experience.
- Nurses who were able to read and write.
- Exclusion criteria:
- Nurses who did not express a willingness to continue participating in the study.
- Nurses with less than one year of work experience.
- Nurses who were not able to read and write.

Data collection involved the utilization of questionnaires with four distinct sections. The initial section concentrated on gathering demographic information, encompassing age, gender, marital status, education level, work experience, and shift patterns. Subsequently, the second to fourth sections encompassed the administration of the Circadian Rhythm Questionnaire, the QWL Questionnaire, and the Nurses’ Productivity Questionnaire, respectively.

The Circadian Rhythm Questionnaire utilized in this study comprises 11 items, each rated on a 5-point Likert scale that covers two independent factors. The first factor is “Flexible/Rigid,” indicating the stability of the circadian rhythm. Individuals who score high on this factor are considered flexible, capable of working in shift systems, and staying awake during atypical times of the day or night. The second factor is “Languid/Vigorous,” representing the range of circadian rhythm. Individuals who score high on this factor are characterized as languid, facing more difficulties with sleepiness and fatigue due to insufficient sleep. This questionnaire consists of two domains: stability of circadian rhythm and range of circadian rhythm. The Cronbach’s alpha coefficients for shift-working nurses’ stability and range domains were 0.70 and 0.82, respectively. The overall reliability of the questionnaire, calculated using Cronbach’s alpha, was 0.76. Jafari-Roodbandi et al. have provided evidence of the scale’s reliability and validity in Iran, confirming its approval with a Cronbach’s α value of 0.76 [24].

Nevertheless, the internal reliability of the questionnaire was assessed using Cronbach’s alpha coefficient, yielding a value of 0.76. Confirmatory Factor Analysis (CFA) was employed to establish the structural validity of the questionnaire. The Kaiser index yielded a value of 0.79, indicating favorable conditions for conducting the factor analysis and confirming the questionnaire’s validity [25].

### The QWL questionnaire

In the present study, the QWL Questionnaire developed by Dehghan-Niri et al. was employed [18]. This questionnaire was specifically designed based on cultural and national models of indigenization. It has five dimensions and employs a 5-point Likert scale for rating responses. The scoring range of the questionnaire spans from 34 to 170, indicating a broad spectrum of possible scores. The questionnaire’s face validity and content validity were established through the participation of nurses, faculty members, and experts, resulting in a content validity index ranging from 0.85 to 1. Furthermore, Dehghan-Niri et al. reported a high-reliability coefficient of 0.91 for the questionnaire, which was determined by calculating Cronbach’s alpha [26].

### Productivity questionnaire

In order to measure productivity, the researchers employed the Productivity Questionnaire developed by Dehghan-Niri et al. [26]. This questionnaire was specifically designed to align with the perspectives of Iranian nurses and was culturally adapted. It comprises 22 items distributed across four sections: effectiveness, efficiency, commitment, and patient accessibility. Respondents rated all items on a 5-point Likert scale. The scoring range for this questionnaire spans from 22 to 110. The content validity index for this questionnaire was reported to range from 0.90 to 1, indicating satisfactory content validity. Moreover, the instrument’s reliability was assessed using Cronbach’s alpha coefficient, yielding a value of 0.84 [26].

### Data analysis

The primary analytical technique employed in this study was SEM. SEM is one of the most potent and suitable methods for analyzing behavioral and social science research data, especially in studies involving multiple variables. The Pearson correlation coefficient was employed to analyze the data and test hypotheses. The R software, version 4.1.1, was utilized to analyze the data and examine the research results.

## Ethical approval and consent to participate

This article results from a part of the dissertation (ethics code IR.QUMS.REC.1402.021) in the Master’s degree. The Ethical Committee of the Qazvin University of Medical Sciences approved all protocols. All methods were carried out in accordance with relevant guidelines and regulations. We provided the participants or their legal guardian(s) with an information sheet, reassured them about anonymity, freedom to withdraw, and confidentiality, explained the purpose of the study, and obtained their informed consent form.

## Results

Ultimately, after distributing 378 questionnaires, approximately 364 completed questionnaires were collected, yielding a response rate of 96%. The findings indicated that out of the 378 participants in this research, 21.16% (80 individuals) were male, while 78.84% (298 individuals) were female employees.

The mean age of the participants was 38.7 ± 9.32. Regarding the educational background, 88.89% of the respondents (336 individuals) had a bachelor’s degree, while 11.11% (42 individuals) had a postgraduate degree. Only 3.03% of the respondents (11 individuals) had fixed shifts, while 96.96% (272) had rotating shifts. Regarding marital status, 61.05% (174 individuals) were married, and 38.95% (111 individuals) were single. The average work experience of the study participants was 6.706 ± 8.476 years. The frequency distribution analysis of the number of children among the study participants revealed that the mean number of children was 0.964 ± 0.634.

The productivity status of nurses was at a desirable level, with an average score of (169.880 ± 11.83). The various dimensions of productivity include effectiveness, efficiency, commitment, and patient accessibility. Among these dimensions, the highest mean score was attributed to efficiency (5.175 ± 27.138), while the lowest mean score was assigned to patient accessibility (2.282 ± 10.505) (Table 1).

**Table 1:** The state of productivity and each of its dimensions in the study subjects

Variable	Lower limit and upper limit	Average	Standard deviation
Effectiveness	5–25	19\531	3/657
Efficiency	7–35	27/138	5/175
Commitment	7–35	25/854	4/957
Patient accessibility	3–15	10/505	2/282
Productivity	22–110	83/169	11/880

Table 2 presents the current state of circadian among nurses. The first factor, denoted as “Flexible/Rigid,” serves as an indicator of circadian rhythm stability. Individuals who achieved high scores on this factor were characterized as flexible, capable of engaging in shift work, and alert during atypical hours, be it during the day or night. The second factor, termed “Languid/Vigorous,” reflects the breadth of the circadian rhythm. However, individuals who attained elevated scores on this factor were classified as languid, as they encountered difficulties in combating sleepiness and lethargy resulting from insufficient sleep for the languid subtype.

**Table 2:** Circadian status and each of its dimensions

Variable	Lower limit and upper limit	Average	Standard deviation
Flexible/Rigid	5–25	13/274	3/732
Languid/Vigorous	6–30	19/597	3/968
Circadian	11–55	32/870	5/219

The range of scores for the change in the circadian ranges from 11 to 55. According to Table 2, the average status of nurses was (219.5 ± 870.32). Individuals had an average status regarding both flexible/rigid and languid/vigorous components. Generally, the circardian status was also average among the sample individuals. Nurses mainly had an inflexible and vigorous status.The average circadian status of nurses was is 219.5 ± 870.32.

The average QWL and its dimensions, including decision-making autonomy, management, interpersonal relationships and support, job-related aspects, salary and benefits, and promotion prospects, were moderate (754.21 ± 994.94), indicating a desirable state. Among the diverse dimensions encompassing QWL, the management-interpersonal relationships, and support emerge as the most favorable conditions, characterized by a noteworthy average score of 865.14 ± 644.44. Conversely, the dimension of salary and benefits exhibits the weakest condition, as evidenced by an average score of 532.2 ± 016.8.

The inferential statistical findings indicated significant differences in the mean between gender and productivity (*p* = 0.008), marital status and productivity (*p* = 0/000), marital status and QWL (*p* = 0.034), and employment status and productivity (*p* = 0.002). Additionally, there was a significant relationship between shift work and QWL (*p* = 0.004) and marital status and QWL (*p* = 0.34). Furthermore, a significant relationship exists between productivity and employment type (*p* = 0.02).

No statistically significant differences were found among the hospitals about productivity, circadian, and QWL (*p* > 0.05).

The results revealed a significant positive correlation between productivity and QWL (*p* < 0.001, Pearson correlation coefficient = 0.22). Furthermore, productivity positively correlated with age, work experience, and the number of children. These findings suggest that as individuals’ age, work experience, and the number of children increases, their productivity level also tends to increase.

The study’s findings reveal a significant positive relationship between QWL and productivity (*p* = 0.037). Furthermore, circadian also exhibits a significant and positive association with QWL (*p* = 0.013). These results suggest that circadian rhythms significantly impact productivity, and the QWL mediates this relationship.

The findings of this study provide evidence that the research model has been validated, as indicated by the favorable goodness-of-fit index RMSEA (RMSEA = 0.079).

## Discussion

This study aimed to investigate the impact of circadian rhythm on the productivity of nurses working in educational hospitals in Qazvin, Iran, with QWL playing a mediating role.

Based on the initial objective of the study, participants exhibited a moderate level of both flexible/rigid and languid/vigorous components. Overall, the circadian rhythm status of the sample population was moderate.

Xiaofu Kang (2022), Lena Yuex (2021), Kyaowang Wang (2022), Zahra Kavousi (2015), and Fatemeh Baghaiepour Sarmi (2016) also investigated the circadian rhythm status among nurses [3, 11, 18, 27, 28]. In Lena Yuex’s (2021) study, the flexibility/rigidity score of nurses working in rotating shifts was 14.4 ± 64.10, while the languid/vigorous score was 80.4 ± 67.17. The results of this study indicated that flexibility was among the influential factors affecting professional effectiveness (*p* = 0.01) [27].

According to Xiaofu Kang (2022), nursing managers can utilize the circadian rhythm variable as a predictive indicator for selecting and assigning individuals to work shifts, aiming to enhance work performance and provide adequate support to employees who are unable to tolerate shift work [27]. The tool used in this study is similar to the one used in the present study. However, one of the positive aspects of our study is the investigation of the impact of two variables, namely productivity and quality of work life, on circadian.

In Kyaowang Wang’s study, blood samples were collected from 30 nurses engaged in rotating shifts. The results indicated that rotating shifts disrupted the circadian rhythm and impacted the regulatory function of B10 immune cells [29]. This study measured the effect of circadian rhythm on physiology. In contrast, our study focuses primarily on the psychological dimensions of circadian and their impact on QWL and productivity.

The second objective of this study was to determine the level of productivity among nurses. Our findings demonstrated that the productivity status among nurses was in a relatively favorable condition. In the research conducted by Hu Geon-Geon (2019), nursing productivity was average (3.3 out of 5) [30]. In Rada Ghadimi’s (2018) study, the average overall score of nursing productivity was 3.92 out of 5, which is above average [31]. These findings align with the current study. Nevertheless, in Amir Jafar Nazari’s (2015) study conducted among nurses in Qom hospitals, nurses reported low productivity [32]. Such differences can be attributed to variations in the study population, sample size, and the tools used in the research.

Our findings revealed a significant relationship between gender and productivity, marital status and productivity, and the type of employment and productivity. From this finding that married individuals had higher productivity, one can infer that marriage leads to better performance in terms of effectiveness, efficiency, commitment, and being available to patients, among the productivity factors. Individuals with formal employment also tend to have a greater sense of security within the organization, which can contribute to their increased productivity.

Besides, productivity positively correlated with age, work experience, and the number of children. This suggests that as individuals’ age, work experience, and number of children increase, their level of productivity also increases. Additionally, in many healthcare educational centers in Iran, their mandatory working hours decrease as nurses’ work experience increases. This can also be a contributing factor to their increased productivity.

Regarding the impact of children on productivity, it can be inferred that individuals who are married and facing infertility issues may experience various psychological problems, which can affect their performance and, consequently, their productivity. On the other hand, individuals who have children are not burdened with infertility concerns and related psychological issues.

In line with our findings, Tufan Agung Akaputra’s (2020) results indicated that gender and length of work experience had a significant relationship with nurse productivity, suggesting a positive correlation between female gender and higher work experience with nurse productivity [33]. The results of this study align with the current study, indicating that female gender and high work experience positively correlate with nurses’ productivity.

In line with our findings, in Hu Geon-Geon’s (2019) study, a group of nurses who were above 36 years old, married, had a master’s degree, regularly worked day shifts, and had experience as a supervisor or head nurse reported better achievements in nursing productivity compared to other nursing groups [30].

Besides, in Park Yunok’s (2018) study, work shift (β = -0.20) and age (β = 0.32) were identified as statistically significant predictive factors for nursing productivity [34].

In the study conducted by Amir Jafar Nazari (2015), a significant correlation (*p* < 0.05) was observed between nursing productivity and various factors, including partnership, career promotion, management of disorders, communication, motivation for work, job security, and job stress [32]. Consistent with the present study, job security exhibited a significant positive correlation with nursing productivity. Moreover, individuals with formal employment demonstrated higher productivity levels [32].

The third objective of this study was to determine the status of QWL among nurses. The mean QWL at the studied hospitals was average, which indicated a relatively favorable condition. In line with our findings, Hong Huo (2020) found the overall mean score of QWL for intensive care unit nurses at a moderate level, and in Fozieh Abadi’s (2017) study, it was at a moderate amount [35, 36].

In the study by Rada Qadimi (2018), the overall QWL score for nurses was 31.2 out of 5, and all dimensions of QWL variables had scores lower than average [31]. In Adel Zahed Babolan’s study, QWL in Ardabil’s hospitals was above average [37]. Such differences can be attributed to different organizational cultures in hospitals, diverse approaches implemented by managers, and varying responsibilities and work environments in different hospitals. The results obtained from Rafiei’s study among nurses showed that the majority of nurses had low QWL [38]. Considering that the study population in both Rafiei’s study and the current study is the same, it can be inferred that QWL among nurses at Qazvin University of Medical Sciences has improved in recent years.

Our findings revealed a meaningful relationship (*P* = 0.004) between the work shift and QWL. Nurses with a fixed work shift had a higher QWL compared to individuals with a work shift. In line with our findings, Mojtaba Karimi (2020) found that individuals with a fixed work shift reported higher productivity [39].

Mohamadian and Vahabzadeh’s (2017) study indicated a high level of work-life conflict in individuals with a rotating shift schedule [40]. In Zahra Kavousi’s (2014) study, compulsory selection of the shift work system reduced job satisfaction [28]. In Hong Huo’s (2020) research, night shift work was among the effective factors affecting the QWL of intensive care unit nurses [35]. Anjana Verma (2018) also concluded that the average scores of nurses working a rotating night shift regarding job satisfaction, sleep, and psychological well-being were significantly lower compared to nurses working day shifts [41]. Farhad Soleimanzadeh (2019) also revealed that the prevalence of high blood pressure in nurses with rotating shifts was higher than in day-working nurses [42]. Pola Fri (2016) also stated that nurses engaged in rotating night shifts had the lowest mean scores for job satisfaction, quality, and quantity of sleep, with chronic recurrent fatigue and psychological and cardiovascular symptoms compared to day shift workers [43].

The fourth objective of the study was to determine the relationship between circadian and QWL among nurses. The fifth objective was to examine the relationship between circadian and productivity. The sixth objective was to investigate the relationship between QWL and productivity among nurses. According to our findings, productivity had a positive and meaningful correlation with QWL (*p* = 0.000) (Pearson correlation coefficient = 0.20). QWL had a meaningful positive relationship with productivity (*p* = 0.37). Circadian rhythm also had a meaningful positive relationship with QWL (*p* = 0.13). These findings suggest that circadian rhythm is effective for productivity through QWL. In line with our findings, Dennis Pera-Jordano (2022) concluded that maintaining a healthy work-life balance for nurses improves QWL and leads to better performance, productivity, and the promotion of safe nursing care [44]. Reem Al-Dossary (2022) also found that QWL, organizational loyalty, and job performance have a positive correlation, and low QWL can negatively impact nurses’ job performance and organizational loyalty [45]. Rafiei (2018) also highlighted the importance of paying attention to QWL and creating suitable contexts to strengthen its dimensions, which can effectively increase nurses’ productivity [38]. Amir Jafar Nazari (2015) also found a meaningful relationship between productivity and nurses’ QWL (*p* = 0.469) [32]. However, Rada Qadimi’s (2018) study was not in line with our findings, and there was no significant correlation between the total score of nurses’ QWL and the total score of their productivity (*p* = 0.15) [31]. Such differences can be attributed to variations in the study population, sample size, and the tools used in the research.

In line with our findings, Tian Feng’s (2021) study showed that night-shift nurses were less active and reported lower life satisfaction than day-shift nurses [6]. In addition, night shift nurses reported lower sleep quality. Wahaj Anwar (2021) also revealed that the night shift is associated with higher stress levels, fatigue, and sleepiness [46].

The results of most studies were in line with our findings in the present study, showing a meaningful positive correlation between nurses’ QWL and their productivity. Besides, individuals’ circadian rhythm and fixed work shifts improve nurses’ QWL.

Based on the findings of this study, the level of productivity among nurses was average. Considering that the QWL directly impacts nurses’ productivity, it is necessary for healthcare managers to pay attention to this issue and develop programs to improve the QWL for nurses. Since the circadian rhythm directly affects the QWL and indirectly affects the productivity of nurses, healthcare officials and policymakers need to prioritize improving this situation. Therefore, it is recommended to consider the circadian rhythm of nurses when planning their shift rotations and consider the factors that can improve their night shift sleep pattern.

It is possible to design applications that incorporate all the factors contributing to individuals’ adaptation to shift work to maximize nurses’ performance and productivity during rotating shifts. Considering that the educational hospitals of Qazvin are specialized, each has different organizational conditions and cultures. Therefore, examining the shift scheduling conditions for nurses in each of these centers is suggested separately, as well as developing plans based on organizational culture and other factors that affect rotating shifts.

**The research limitations are as follows**:

This is the first kind of study conducted in Iran. However, it had some limitations. Firstly, time constraints for completing the questionnaires by nurses: Healthcare workers, especially nurses, are often busy due to the high volume of activities and staff shortages. They may not have the necessary interest and attention to complete the questionnaires. To address this issue, efforts were made to coordinate with the nurses in advance so that they had sufficient time to complete the questionnaires. Besides, incomplete questionnaire completion by nurses: A 10% drop rate has been considered from the original sample size to account for incomplete or unusable questionnaires. This ensures deficiencies or unusable questionnaires do not affect the required sample size. In addition, generalizability to non-academic and non-governmental hospitals: Since this study was conducted among nurses in educational hospitals, caution should be exercised when generalizing the results to non-academic and non-governmental hospitals.

Suggestions for future research:

- Conducting this study among other healthcare groups, such as paramedics and physicians.
- Conducting a study that examines the factors affecting productivity in rotating shifts among nurses.
- Conducting a study that investigates the relationship between shift work and the quality of life of nurses.
- Conducting a study that examines the impact of the alignment between work shift and circadian rhythm on the productivity of nurses.

## Conclusion

The findings of this study indicated a significant and positive relationship between nurses’ QWL and their productivity. Additionally, a high QWL was significantly associated with job satisfaction. Considering that nurses’ performance significantly impacts patients’ treatment processes, improving their performance can benefit patients. One of the factors that improves nurses’ productivity is increasing their QWL. Based on this study, nurses’ circadian rhythm positively correlates with their QWL. Therefore, utilizing methods and programs to coordinate nurses’ circadian conditions will improve performance and productivity. Nursing managers should consider nurses’ circadian rhythms when planning their shift schedules.

## Data Availability

The datasets used and/or analyzed during the current study available from the corresponding author on reasonable request. The entire dataset is in Farsi language. The Data can be available in English language for the readers and make available from the corresponding author on reasonable request.
